# Association between hemoglobin within the normal range and hemoglobin A1c among Chinese non-diabetes adults

**DOI:** 10.1186/s12902-021-00704-x

**Published:** 2021-02-27

**Authors:** Yi Lai, Zhihong Lin, Zhongxin Zhu

**Affiliations:** 1 Department of Emergency, Xiaoshan Affiliated Hospital of Wenzhou Medical University, Hangzhou, 311200 Zhejiang China; 2 Department of General Surgery, Xiaoshan Affiliated Hospital of Wenzhou Medical University, Hangzhou, 311200 Zhejiang China; 3 Department of Osteoporosis Care and Control, Xiaoshan Affiliated Hospital of Wenzhou Medical University, Hangzhou, 311200 Zhejiang China; 4 Clinical Research Center, Xiaoshan Affiliated Hospital of Wenzhou Medical University, Hangzhou, 311200 Zhejiang China

**Keywords:** Hemoglobin, Hemoglobin A1c, Diabetes, Biomarker, China health and nutrition survey

## Abstract

**Background:**

Hemoglobin A1c (HbA1c) is the product of a non-enzymatic chemical reaction between hemoglobin (Hb) and glucose. However, the association between Hb and HbA1c remains to be fully elucidated in view of the controversial findings reported to date. Therefore, our aim in this study was to evaluate the association between Hb levels within the normal range and HbA1c levels among Chinese non-diabetes adults using cross-sectional data from the China Health and Nutrition Survey 2009.

**Methods:**

Our analysis was based on the data of 1659 non-diabete adults 20–49 years of age. Multivariable linear models were applied to examine the association between Hb and HbA1c levels. Subgroup analyses stratified by age and sex were also performed.

**Results:**

The association between Hb and HbA1c levels was positive in the unadjusted model (β =0.020, 95% CI: 0.008, 0.032). However, this association did not remain significant when the regression model was minimally adjusted for age and sex (β =0.006, 95% CI: − 0.014, 0.024); this association became negative when the model was further adjusted for covariates whose effect estimates of HbA1c levels more than 10% (β = − 0.042, 95% CI: − 0.064, − 0.020). The association remained negative on subgroup analyses stratified by age (20–34 years: β = − 0.052, 95% CI: − 0.091, − 0.013; 35–49 years: β = − 0.041, 95% CI: − 0.068, − 0.014) and sex (men: β = − 0.042, 95% CI: − 0.074, − 0.010; women: β = − 0.042, 95% CI: − 0.073, − 0.012) when controlling for covariates.

**Conclusions:**

Our findings revealed that Hb levels within the normal range were negatively associated with HbA1c levels among Chinese non-diabetes adults. Confounding factors, such as red blood cell counts can affect the association between Hb and HbA1c levels.

**Supplementary Information:**

The online version contains supplementary material available at 10.1186/s12902-021-00704-x.

## Background

Diabetes is one of the most prevalent diseases globally. Almost 10% of the world’s adult population (nearly 500 million individuals) live with diabetes, with the prevalence expected to increase to 700 million by 2045 [1]. Moreover, although the prevalence of diabetes is high, nearly half of the individuals living with diabetes are unaware of their disease status [2]. If left untreated, diabetes is associated with devastating short- and long-term complications [3]. As early interventions might delay or even prevent full-blown diabetes, it is important to identify individuals at high risk of diabetes. Accordingly, ongoing studies are assessing the utility of novel and less studied biomarkers of diabetes [4].

Hemoglobin (Hb), a protein only found in red blood cells, becomes glycated or coated with glucose from the bloodstream. As the extent of Hb glycation is influenced by the amount of blood glucose, increased blood glucose levels are reflected on the surface of the Hb protein [5]. Hemoglobin A1c (HbA1c) is the product of a non-enzymatic chemical reaction between Hb and glucose [6]. The HbA1c level reflects the average level of blood glucose over the past 90 days [7]. As a measure of blood glucose, HbA1c has several advantages over fasting plasma glucose generally used in practice: greater convenience as no fasting is required; greater pre-analytical stability; and less day-to-day variability due to stress, diet, or illness [8]. However, previous studies that focused on the relationship between Hb and HbA1c in patients with anemia yielded inconsistent findings [9]. Moreover, the association between Hb levels within the normal range and HbA1c levels remains to be clarified. Therefore, our aim in this study was to evaluate the association between Hb levels within the normal range and HbA1c levels among Chinese non-diabetes adults using cross-sectional data from the China Health and Nutrition Survey (CHNS).

## Methods

### Study population

The CHNS is an ongoing, open, population-based study conducted by the National Institute for Nutrition and Food Safety at the Chinese Center for Disease Control and Prevention (CCDC) and the Carolina Population Center at the University of North Carolina at Chapel Hill (UNC), designed to examine the health and nutrition status of the Chinese population [10]. In 2009, the Department of Laboratory Medicine of China-Japan Friendship Hospital, Ministry of Health joined this project as the lead agency for the collection, storage and analysis of biospecimens. The CHNS protocols were approved by the Institutional Review Board of National Institute of Nutrition and Food Safety at Chinese Center for Disease Control and Prevention, the Carolina Population Center at the University of North Carolina at Chapel Hill, and the China-Japan Friendship Hospital, Ministry of Health, China. All participants provided written informed consent for the use of their data in research.

**Fig. 1:**
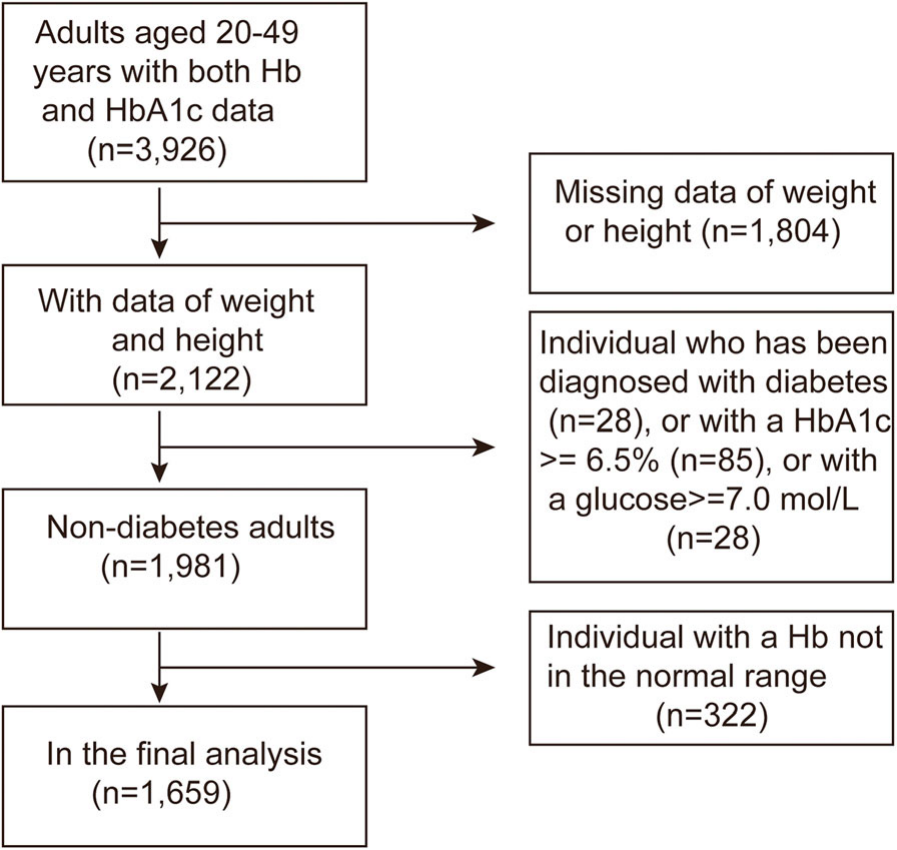
Flow chart of the participants

A total of 10 waves of the survey have been conducted since 1989. Notably, blood samples were collected as part of the CHNS for the first time in the 2009 survey. In this study, 3926 adults aged 20–49 years, with available data on both Hb and HbA1c levels were deemed eligible. We excluded individuals with missing weight or height data (*n* = 1804); a diagnosis of diabetes (*n* = 28), an HbA1c level ≥ 6.5% (*n* = 85), or glucose level ≥ 7.0 mol/L (*n* = 28); and a Hb level outside the normal range (*n* = 322). The data for the remaining 1659 non-diabetes adults were included in our final analysis (Fig. 1).

### Study variables

In the CHNS, blood samples (12 mL) were collected after overnight fasting for > 8 hours, and analyzed under strict quality control at the Beijing central laboratory as per previously described methods, using a 3- or 5-part classification automated hematology analyzer (Hitachi 7600 automated analyzer, Hitachi Inc., Japan) [11].

The Hb level, considered as the variable of exposure in our study, was determined by the volume, conductivity, and scatter method for blood cell analysis using the LH750 hematology analyzer (Beckman Coulter, USA). The normal range for Hb levels is 13.3–17.7 g/dL for adult men and 11.7–15.7 g/dL for adult women [12]. The HbA1c level, defined as the outcome variable, was measured using high-performance liquid chromatography using a National Glycohemoglobin Standardization Program certified automated analyzer (model HLC-723 G7, Japan). The level of fasting plasma glucose was measured using the GOD-PAP method (Randox Laboratories Ltd., UK). Diabetes was defined as an HbA1c level ≥ 6.5% or a fasting plasma glucose level ≥ 7.0 mmol/L, following the 2019 American Diabetes Association criteria [13].

Data on the following covariates were extracted for inclusion in our analysis: age, sex, ethnicity, education level, activities (based on the question “How much do you like to participate in this activity”), smoking behavior, alcohol consumption, body mass index (BMI), total protein, total cholesterol, blood glucose, alanine aminotransferase (ALT), uric acid, serum creatinine, white blood cell (WBC) count, red blood cell (RBC) count, and platelet (PLT) count. Details regarding the acquisition process of data on Hb levels, HbA1c, and other covariates have been previously described in detail [14].

### Statistical analysis

All analyses were performed using statistical software R (version 3.4.3) and EmpowerStats (X&Y Solutions, Boston, MA). Between-group differences were evaluated using one-way ANOVA (normal distribution) and Kruskal-Wallis H (skewed distribution) tests for continuous variables and a chi-squared test for categorical variables. The association between HbA1c and Hb levels was evaluated using multivariate linear regression analyses, with the creation of three models: an unadjusted model; minimally adjusted model, controlling for age and sex; and a fully adjusted model, controlling for age, sex, smoking behavior, alcohol consumption, BMI, blood glucose, WBC count, and RBC count. We selected these confounders on the basis of their effect estimates of more than 10%. Supplementary Table 1 shows the associations of each covariate with HbA1c. The Hb level was categorized into quartiles; a sensitivity analysis as performed, and the *P*-value of the trend was calculated. Next, we performed subgroup analyses stratified by age and sex. We further used generalized additive models and smooth curve fittings to explore potential non-linearity in the association between Hb and HbA1c levels. A *P*-value < 0.05 was considered significant for these analyses.

## Results

Baseline characteristics for our study group, which included 781 men and 878 women, are presented in Table 1. Men had higher levels of education, and a higher BMI than women. Men had higher total cholesterol, blood glucose, ALT, uric acid, serum creatinine, WBC, RBC, Hb, and HbA1c values but lower total protein, and PLT values. Furthermore, the proportions of men who liked activity, smoked, and consumed alcohol were higher than those of women (*p* < 0.05 for each).

**Table 1:** The characteristics of participants

	Men (*n* = 781)	Women (*n* = 878)	*P* value
Age (years)	37.70 ± 8.01	38.07 ± 7.67	0.335
Ethnicity (%)			0.883
Han population	92.45	92.60	
Non- Han population	6.79	6.83	
Not recorded	0.77	0.57	
Education level (%)			< 0.001
None or grad from primary	14.85	26.54	
Middle school degree	60.82	52.73	
More than middle school	18.82	15.95	
Not recorded	5.51	4.78	
Activities (%)			< 0.001
Dislike	59.41	67.08	
Neutral	21.00	21.87	
Like	19.59	11.05	
Smoking behavior (%)			< 0.001
No	38.41	97.95	
Yes	61.59	2.05	
Alcohol consumption (%)			< 0.001
No	32.78	91.23	
Yes	67.22	8.77	
Body mass index (kg/m^2^)	23.92 ± 3.33	23.38 ± 3.32	< 0.001
Total protein (g/L)	76.21 ± 4.65	77.06 ± 4.79	< 0.001
Total cholesterol (mmol/L)	4.66 ± 0.93	4.46 ± 0.86	< 0.001
Blood glucose (mmol/L)	5.00 ± 0.61	4.92 ± 0.55	0.004
Alanine aminotransferase (U/L)	29.52 ± 21.56	19.83 ± 21.57	< 0.001
Uric acid (umol/L)	347.59 ± 90.04	232.58 ± 60.62	< 0.001
Serum creatinine (umol/L)	91.66 ± 10.97	74.07 ± 8.73	< 0.001
White blood cell count (10^9^/L)	6.63 ± 1.77	6.20 ± 1.86	< 0.001
Red blood cell count (10^12^/L)	5.09 ± 0.43	4.50 ± 0.40	< 0.001
Platelet count (10^9^/L)	222.33 ± 60.93	233.84 ± 57.74	< 0.001
Hemoglobin (g/dL)	15.60 ± 0.95	13.37 ± 0.88	< 0.001
Hemoglobin A1c (%)	5.51 ± 0.37	5.45 ± 0.36	< 0.001

Mean ± SD for continuous variables: *P* value was calculated by one-way ANOVA (normal distribution) and Kruskal-Wallis H (skewed distribution) test

% for categorical variables: *P* value was calculated by chi-square test

The association between Hb and HbA1c levels was positive in the unadjusted model (β =0.020, 95% CI: 0.008, 0.032). However, this association was no longer significant after controlling for age and sex in the minimally adjusted model (β =0.006, 95% CI: − 0.014, 0.024), and became negative in the fully adjusted model (β = − 0.042, 95% CI: − 0.064, − 0.020), with a P for trend of < 0.001. The results are presented in Table 2.

**Table 2:** Association between hemoglobin level (g/dL) and hemoglobin A1c level (%)

	Unadjusted model β (95% CI)	Minimally adjusted model β (95% CI)	Fully adjusted model β (95% CI)
Hemoglobin	0.020 (0.008, 0.032)^**^	0.006 (− 0.014, 0.024)	−0.042 (− 0.064, − 0.020)^***^
Hemoglobin (Quartile)			
Q1	Reference	Reference	Reference
Q2	0.03 (−0.02, 0.08)	0.02 (−0.03, 0.07)	− 0.03 (− 0.08, 0.02)
Q3	0.07 (0.02, 0.12)	0.02 (− 0.04, 0.09)	−0.07 (− 0.14, − 0.01)
Q4	0.07 (0.02, 0.12)	0.01 (− 0.07, 0.08)	−0.14 (− 0.22, − 0.06)
P for trend	0.002	0.771	< 0.001

Unadjusted model: no covariates were adjusted

Minimally adjusted model: age and sex were adjusted

Fully adjusted model: age, sex, smoking behavior, alcohol consumption, body mass index, blood glucose, white blood cell count, and red blood cell count were adjusted

^*^ *P* < 0.05, ^**^ *P* < 0.01, ^***^ *P* < 0.001

**Fig. 2:**
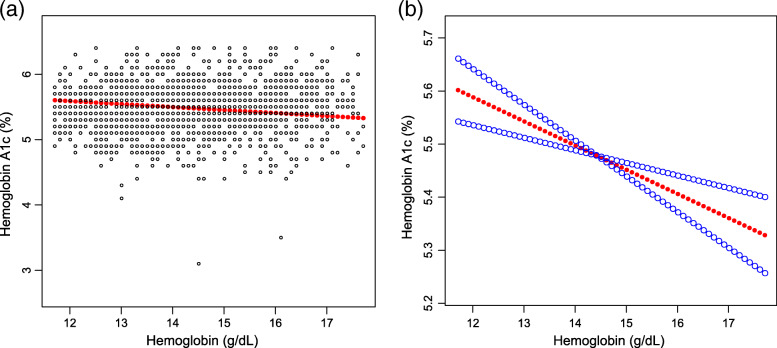
The association between hemoglobin and hemoglobin A1c. **a** Each black point represents a sample. **b** Solid rad line represents the smooth curve fit between variables. Blue bands represent the 95% of confidence interval from the fit. Age, sex, smoking behavior, alcohol consumption, body mass index, blood glucose, white blood cell count, and red blood cell count were adjusted

**Fig. 3:**
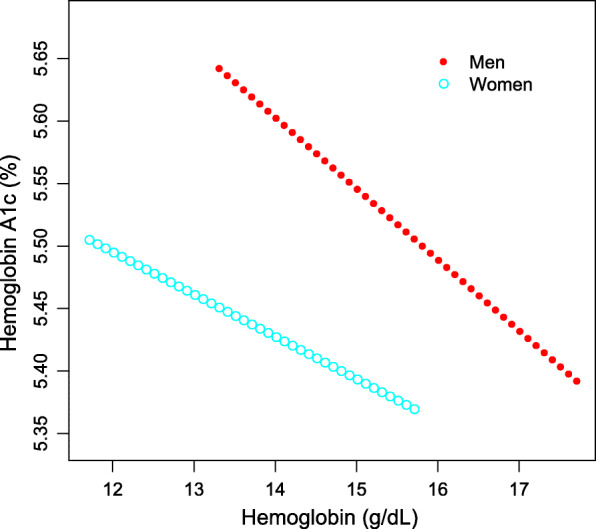
The association between hemoglobin and hemoglobin A1c, stratified by sex. Age, smoking behavior, alcohol consumption, body mass index, blood glucose, white blood cell count, and red blood cell count were adjusted

**Fig. 4:**
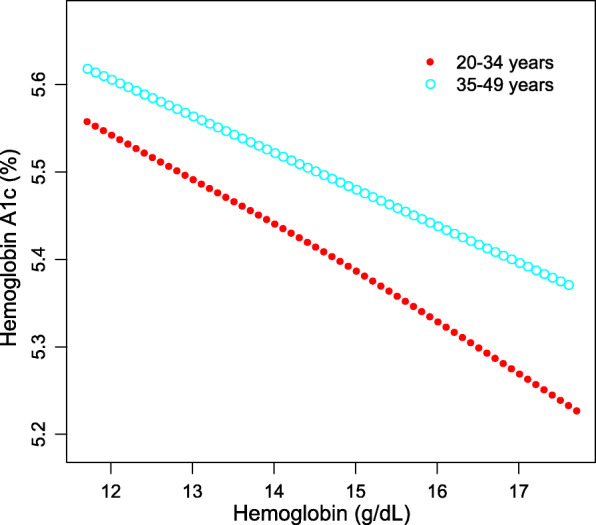
The association between hemoglobin and hemoglobin A1c, stratified by age. Age, sex, smoking behavior, alcohol consumption, body mass index, blood glucose, white blood cell count, and red blood cell count were adjusted

As shown in Table 3, after controlling for potential confounding factors, the association between Hb and Hb A1c levels remained negative in the subgroup analyses stratified by age (20–34 years: β = − 0.052, 95% CI: − 0.091, − 0.013; 35–49 years: β = − 0.041, 95% CI: − 0.068, − 0.014) and sex (men: β = − 0.042, 95% CI: − 0.074, − 0.010; women: β = − 0.042, 95% CI: − 0.073, − 0.012). The results of generalized additive models and smooth curve fittings further confirmed this inverse associaton between Hb and HbA1c levels (Figs. 2, 3, 4).

**Table 3:** Association between hemoglobin (g/dL) and hemoglobin A1c (%), stratified by age and sex

	Unadjusted model β (95% CI)	Minimally adjusted model β (95% CI)	Fully adjusted model β (95% CI)
Stratified by age			
20–34 years	0.009 (−0.011, 0.030)	− 0.011 (− 0.043, 0.022)	−0.052 (− 0.091, − 0.013)^**^
35–49 years	0.025 (0.010, 0.040)^**^	0.012 (− 0.011, 0.036)	−0.041 (− 0.068, − 0.014)^**^
Stratified by sex			
Men	− 0.006 (− 0.033, 0.022)	−0.005 (− 0.032, 0.023)	−0.042 (− 0.074, − 0.010)^**^
Women	0.021 (− 0.006, 0.047)	0.016 (− 0.011, 0.043)	−0.042 (− 0.073, − 0.012)^**^

Unadjusted model: no covariates were adjusted

Minimally adjusted model: age and sex were adjusted

Fully adjusted model: age, sex, smoking behavior, alcohol consumption, body mass index, blood glucose, white blood cell count, and red blood cell count were adjusted

Sex is not adjusted in the subgroup analysis stratified by sex

^*^ *P* < 0.05, ^**^ *P* < 0.01, ^***^ *P* < 0.001

## Discussion

In this study, we evaluated a large sample of Chinese non-diabetes adults to explore the association between Hb levels within the normal range and HbA1c levels. Our results showed that Hb levels were independently and negatively associated with HbA1c levels in both men and women.

Anemia is a common condition, clinically defined as a hematocrit value or blood Hb level that is below the normal range. Anemia is often associated with diabetes and is known to increase the risk of diabetes-related complications [15]. However, previous studies that investigated the association between Hb and HbA1c levels in patients with anemia have yielded contradictory conclusions. While several studies have reported that HbA1c level was significantly higher in individuals with anemia [16–18], others did not identify a difference in HbA1c levels between individuals with anemia and healthy controls [19], or found a lower HbA1c level in patients with anemia [20].

Recently, Grossman et al. [21] conducted a large retrospective cohort study including 11,352 elderly non-diabetic community individuals to evaluate the association between Hb and HbA1c levels. Although they found that Hb levels were significantly lower among individuals in the highest HbA1c quintile than among individuals in the other quintiles, the correlation between HbA1c and Hb levels was negligible. In our study, the correlation between Hb levels in the normal range and HbA1c levels was positive in the unadjusted model but became non-significant after adjusting for age and sex and became negative after adjustment for potential confounding factors. Differences in the reported association between Hb and HbA1c levels between studies might reflect the differences in study design and confounding variables used for adjustment.

The HbA1c test is easy to perform, making it widely available as an ideal tool for busy primary care and endocrine practices [22]. However, the HbA1c level is impacted by numerous factors, such as race, RBC disorders, and hemoglobinopathies [23]. Moreover, iron deficiency anemia may cause a spurious increase in HbA1c values [24]. Our findings supported an independent negative association between Hb and HbA1c levels among non-diabetes adults with normal Hb levels, with this relationship being stable in both men and women.

The main strength of our study was the large-scale, and population-based study design; approximately 56% of the Chinese population is covered by the CHNS. Therefore, our results can be generalized to the entire Chinese population. The limitations of our study also need to be acknowledged. First, the CHNS provides cross-sectional data, and therefore, the causal relationship between Hb and HbA1c cannot be determined. A longitudinal study is warranted in this regard. Second, we excluded individuals with diabetes, with abnormal Hb values, and an age > 50 years. Therefore, our conclusions are not generalizable to these populations. Third, although we adjusted for several important potential confounders, other potential confounding factors, including hemoglobinopathies, iron deficiency, drug use, menstruation status, hypothyroidism, and chronic kidney disease, may have introduced bias. The results of our study differed according to the model used, as the association between Hb and HbA1c levels was affected by the choice of the covariates included in the model, such as the RBC count. However, conditions such iron deficiency and altered RBC lifespan, which may also have important effects on the association between Hb and HbA1c levels, were not included in the CHNS surveys. Therefore, further large sample prospective studies that include measurements of these additional variables are warranted.

## Conclusions

Our findings revealed that Hb levels within the normal range were negatively associated with HbA1c levels among Chinese non-diabetes adults. Confounding factors, such as RBC counts, can affect the association between Hb and HbA1c levels.

## Supplementary Information

**Additional file 1: Table S1.** Univariate analysis of the associations of each covariate with HbA1c.

## Data Availability

Details of the study design, sampling strategies and data are available at the World Wide Web site (https://www.cpc.unc.edu/projects/china). The datasets used and/or analysed during the current study are available from the corresponding author on reasonable request.

## Abbreviations

Hb: Hemoglobin
HbA1c: Hemoglobin A1c
CHNS: the China Health and Nutrition Survey
CCDC: the Chinese Center for Disease Control and Prevention
UNC: the University of North Carolina at Chapel Hill
BMI: Body mass index
ALT: Alanine aminotransferase
WBC: White blood cell
RBC: Red blood cell
PLT: Platelet
