# Supplementary material for: Association between hemoglobin within the normal range and hemoglobin A1c among Chinese non-diabetes adults
Source: BMC Endocr Disord. 2021 Feb 27;21:35. doi: 10.1186/s12902-021-00704-x (PMC7916310; doi:10.1186/s12902-021-00704-x)
Supplement: Supplementary file 1 — Additional file 1: Table S1. Univariate analysis of the associations of each covariate with HbA1c. [file 12902_2021_704_MOESM1_ESM.doc]

Supplementary table 1 Univariate analysis of the associations of each covariate with HbA1c.

| HbA1c | Statistics | β (95% CI), P value |
| --- | --- | --- |
| Age (years) | 37.9 ± 7.8 | 0.006 (0.004, 0.008) <0.001 |
| Sex |  |  |
| Men | 781 (47.1%) | Reference |
| Women | 878 (52.9%) | -0.064 (-0.099, -0.028) <0.001 |
| Ethnicity (%) |  |  |
| Han population | 1535 (92.5%) | Reference |
| Non- Han population | 113 (6.8%) | -0.12 (-0.19, -0.06) <0.001 |
| Not recorded | 11 (0.7%) | 0.12 (-0.09, 0.34) 0.264 |
| Education level (%) |  |  |
| None or grad from primary | 349 (21.0%) | Reference |
| Middle school degree | 938 (56.5%) | 0.032 (-0.012, 0.077) 0.155 |
| More than middle school | 287 (17.3%) | -0.043 (-0.100, 0.013) 0.133 |
| Not recorded | 85 (5.1%) | 0.23 (0.15, 0.32) <0.001 |
| Activities (%) |  |  |
| Dislike | 1053 (63.5%) | Reference |
| Neutral | 356 (21.5%) | 0.058 (0.014, 0.102) 0.009 |
| Like | 250 (15.1%) | -0.059 (-0.109, -0.008) 0.022 |
| Smoking behavior (%) |  |  |
| No | 1160 (69.9%) | Reference |
| Yes | 499 (30.1%) | 0.084 (0.045, 0.122) <0.001 |
| Alcohol consumption (%) |  |  |
| No | 1057 (63.7%) | Reference |
| Yes | 602 (36.3%) | 0.059 (0.022, 0.095) 0.002 |
| Body mass index (kg/m^2^) | 23.6 ± 3.3 | 0.024 (0.019, 0.029) <0.001 |
| Total protein (g/L) | 76.7 ± 4.7 | -0.001 (-0.005, 0.003) 0.574 |
| Total cholesterol (mmol/L) | 4.6 ± 0.9 | 0.060 (0.040, 0.079) <0.001 |
| Blood glucose (mmol/L) | 5.0 ± 0.6 | 0.16 (0.13, 0.19) <0.001 |
| Alanine aminotransferase (U/L) | 24.4 ± 22.1 | 0.0013 (0.0005, 0.0021) 0.002 |
| Uric acid (umol/L) | 286.6 ± 95.1 | 0.0003 (0.0001, 0.0005) 0.003 |
| Serum creatinine (umol/L) | 82.3 ± 13.2 | 0.0015 (0.0002, 0.0028) 0.028 |
| White blood cell count (10^9^/L ) | 6.4 ± 1.8 | 0.022 (0.012, 0.032) <0.001 |
| Red blood cell count (10^12^/L ) | 4.8 ± 0.5 | 0.088 (0.053, 0.122) <0.001 |
| Platelet count (10^9^/L ) | 228.4 ± 59.5 | 0.0006 (0.0003, 0.0008) <0.001 |
